# Elimination of radiation‐induced senescent cancer cells and stromal cells in vitro by near‐infrared photoimmunotherapy

**DOI:** 10.1002/cam4.7381

**Published:** 2024-06-18

**Authors:** Motofumi Suzuki, Hisataka Kobayashi, Daiki Hara, Hirofumi Hanaoka

**Affiliations:** ^1^ Division of Fundamental Technology Development Near InfraRed Photo‐ImmunoTherapy Research Institute at Kansai Medical University Hirakata Osaka Japan; ^2^ Molecular Imaging Branch, Center for Cancer Research National Cancer Institute, National Institutes of Health Bethesda Maryland USA

**Keywords:** molecular target therapy, near‐infrared photoimmunotherapy, senescent cells, tumor stroma, tumor treatment

## Abstract

**Introduction:**

Therapy‐induced senescent cancer and stromal cells secrete cytokines and growth factors to promote tumor progression. Therefore, senescent cells may be novel targets for tumor treatment. Near‐infrared photoimmunotherapy (NIR‐PIT) is a highly tumor‐selective therapy that employs conjugates of a molecular‐targeting antibody and photoabsorber. Thus, NIR‐PIT has the potential to be applied as a novel senolytic therapy. This study aims to investigate the efficacy of NIR‐PIT treatment on senescent cancer and stromal cells.

**Methods:**

Two cancer cell lines (human lung adenocarcinoma A549 cells and human pancreatic cancer MIA PaCa‐2 cells) and two normal cell lines (mouse fibroblast transfected with human epidermal growth factor receptor 2 [HER2] cells and human fibroblast WI38 cells) were used. The cytotoxicity of NIR‐PIT was evaluated using anti‐epidermal growth factor receptor (EGFR) antibody panitumumab and anti‐HER2 antibody transtuzumab.

**Results:**

Cellular senescence was induced in A549 and MIA PaCa‐2 cells by 10 Gy *γ*‐irradiation. The up‐regulation of cellular senescence markers and characteristic morphological changes in senescent cells, including enlargement, flattening, and multinucleation, were observed in cancer cells after 5 days of *γ*‐irradiation. Then, NIR‐PIT targeting EGFR was performed on these senescent cancer cells. The NIR‐PIT induced morphological changes, including bleb formation, swelling, and the inflow of extracellular fluid, and induced a significant decrease in cellular viability. These results suggested that NIR‐PIT may induce cytotoxicity using the same mechanism in senescent cancer cells. In addition, similar morphological changes were also induced in radiation‐induced senescent 3T3‐HER2 fibroblasts by NIR‐PIT targeting human epidermal growth factor receptor 2.

**Conclusion:**

NIR‐PIT eliminates both senescent cancer and stromal cells in vitro suggesting it may be a novel strategy for tumor treatment.

## Graphical abstract

Since therapy‐induced senescent cells secrete cytokines and growth factors to promote tumor progression, senescent cells may be novel targets for tumor treatment. In the current study, novel senolysis using near‐infrared photoimmunotherapy, which is a highly selective cancer treatment, was developed.

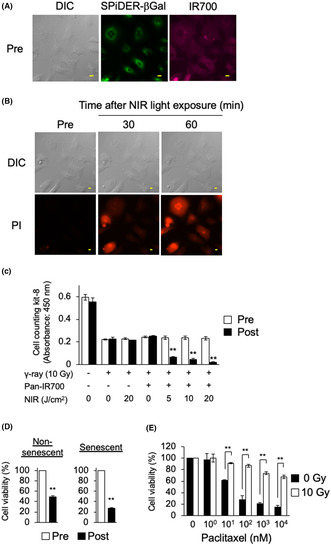

## 1 INTRODUCTION

Conventional anti‐cancer treatments such as chemotherapy and radiotherapy induce cellular senescence in tumor tissue. Of tumors of patients who did not undergo chemotherapy, only 10% stained positive for senescence‐associated beta‐galactosidase (SA‐β‐gal), a senescence marker, whereas the rate was as high as 41% of tumors of patients who received adjuvant chemotherapy.^1^ Cellular senescence is a phenomenon of irreversible cell‐cycle arrest. The characteristics of senescent cells include having a flattened and enlarged morphology, and a senescence‐associated secretory phenotype (SASP) that secretes cytokines and growth factors that can affect surrounding cells. Therapy‐induced senescence has both beneficial and noxious effects in tumor control. Permanent cell‐cycle arrest by senescence sometimes contributes to the good outcome of anti‐cancer therapy.^2^, ^3^ Therefore, several kinase inhibitors can potentiate the therapeutic effects of anti‐cancer therapy by increasing cellular senescence.^4^, ^5^ However, therapy‐induced senescence can also promote adverse effects in chemotherapy as well as aggressive relapses and secondary tumors.^6^, ^7^ The SASP of senescent melanoma induced by cisplatin promotes non‐senescent melanoma cell growth via the ERK1/2‐RSK1 pathway.^8^ Accordingly, growth arrest by cellular senescence acts as a temporary tumor suppressor; however, the long‐term presence of senescent cells is detrimental to tumor control. Thus, senescent cells can be novel targets in tumor treatment.

Tumor tissue is heterogeneous and consists of not only cancer cells but also various stromal cells, such as fibroblasts, immune cells, and endothelial cells. Notably, tumor‐surrounding fibroblasts are referred to as cancer‐associated fibroblasts (CAFs). Such CAFs are a central component of the tumor stroma in both primary and metastatic tumors.^9^ The CAFs secrete various cytokines, growth factors, and chemokines and promote tumor progression, invasion, and metastasis.^10^ Previous reports suggest that CAFs contribute to the radioresistance of cancer cells through interleukin‐6 and transforming growth factor‐β signaling pathways.^11^, ^12^, ^13^ Moreover, residual CAFs after radiotherapy are prone to being senescent cells; thus, senescent stroma cells can also be a promising target for tumor treatment.

To date, various senolytic compounds have been reported, such as BCL‐XL/BCL‐2 inhibitors, HSP90 inhibitors, p53 pathway modulators, and natural products.^14^, ^15^ Previous reports suggest that navitoclax (ABT‐263) eliminates senescent cells induced by chemotherapy or radiotherapy, and consequently reduces cancer recurrence and metastasis.^6^, ^16^ However, many senolytic agents do not selectively eliminate senescent cells and are cytotoxic to normal cells. Indeed, navitoclax induces apoptosis of circulating platelets and leads to thrombocytopenia and neutropenia in patients with small‐cell lung cancer and relapsed or refractory lymphoid malignancies.^17^, ^18^, ^19^ In addition, a dasatinib plus quercetin cocktail, the most well‐studied senolytic agent, is not universally effective against senescent cells, and is ineffective against the senescent hepatocellular carcinoma cells induced by doxorubicin.^20^ Therefore, the development of a novel senolytic strategy with fewer adverse effects and higher therapeutic efficiency is desired.

Near‐infrared photoimmunotherapy (NIR‐PIT) is a recently developed highly tumor‐selective therapy that employs conjugates of a molecular‐targeting antibody and photoabsorber (IRDye700DX; IR700), which absorbs 690‐nm light in the NIR range.^21^, ^22^ These conjugates and NIR light are harmless, and cytotoxicity is induced only when NIR light is irradiated while the conjugates are bound to an antigen on target cells. The potential for human safety and the therapeutic efficacy of NIR‐PIT targeting the epidermal growth factor receptor (EGFR) has been reported in patients with head and neck squamous cell carcinoma.^23^, ^24^ In NIR‐PIT, the photochemical reaction of IR700 on the cellular membrane causes severe membrane injury, leading to cell death. Therefore, NIR‐PIT has the potential to induce cytotoxic effects in all types of cells if target antigens are expressed. Indeed, NIR‐PIT targeting various antigens on a variety of cells has been demonstrated to have high therapeutic efficacy.^22^, ^25^ Thus, NIR‐PIT could be applied to the elimination of senescent cells in a tumor, but no reports exist of studies on senescent cells. NIR‐PIT has great potential as a novel senolytic strategy with fewer adverse effects and higher therapeutic efficiency. In the current study, we sought to investigate the efficacy of NIR‐PIT targeting EGFR and human epidermal growth factor receptor type‐2 (HER2) in senescent cancer and senescent stromal cells induced by *γ*‐irradiation in vitro.

## 2 MATERIALS AND METHODS

### 2.1 Reagents and antibodies

A water‐soluble, silicon‐phthalocyanine derivative, IRDye700DX N‐hydroxysuccinimide ester (IR700‐NHS) was purchased from Li‐COR Bioscience (Lincoln, NE, USA). Panitumumab, a humanized IgG2 monoclonal antibody directed against EGFR was obtained from Amgen (Thousand Oaks, CA, USA). Trastuzumab, a humanized IgG1 monoclonal antibody directed against HER2, was obtained from Chugai Pharmaceutical (Tokyo, Japan). Anti‐EGFR, anti‐p21, and anti–gamma H2A.X (phospho S139) antibodies were obtained from Abcam (Cambridge, UK). Anti–thymidine kinase 1 (TK1) and anti‐beta‐actin antibodies were purchased from Proteintech (Rosemont, IL, USA). All other chemicals used were of reagent grade.

### 2.2 Synthesis of IR700‐conjugated panitumumab and trastuzumab

Panitumumab or trastuzumab (1 mg, 6.8 nmol) was incubated with IR700‐NHS (60.2 μg, 30.8 nmol) in 0.1 M Na_2_HPO_4_ (pH 8.5) at room temperature overnight. The mixture was purified using a Sephadex G25 column (PD‐10; GE Healthcare, Piscataway, NJ, USA) to obtain IR700‐conjugated panitumumab and trastuzumab (Pan‐IR700 and Tra‐IR700, respectively). The protein concentrations were determined with protein assay BCA reagent (Fujifilm Wako Pure Chemical Industries; Osaka, Japan) by measuring the light absorption at 562 nm with a spectrophotometer (V‐730Bio; JASCO, Tokyo, Japan). The concentration of IR700 was measured by absorption at 689 nm with a spectrophotometer to determine the number of fluorophore molecules conjugated to antibodies. Each synthesis was controlled so that an average of three IR700 molecules were bound to a single antibody.

### 2.3 Cell culture

The human lung cancer cell line, A549, and the human normal fibroblast cell line, WI‐38, were obtained from RIKEN Cell Bank (Tsukuba, Japan). The human pancreatic cancer cell line, MIA PaCa‐2, was obtained from the Cell Resource Center for Biomedical Research, Institute of Development, Aging and Cancer, Tohoku University (Sendai, Japan). A mouse fibroblast‐like cell line NIH/3T3, highly expressing human HER2 (3 T3‐HER2), was established by Dr. Kobayashi's laboratory. All cells were maintained in Dulbecco's modified Eagle's medium (DMEM; Fujifilm Wako Pure Chemical Industries) or RPMI‐1640 medium (Fujifilm Wako Pure Chemical Industries) supplemented with 10% (v/v) fetal bovine serum (FBS; GE Healthcare, South Lagan, UT) and antibiotics (100 μg/mL penicillin and streptomycin) at 37°C in a humidified atmosphere of 5% CO_2_.

### 2.4 Induction and detection of senescent cells

Cells were seeded into a culture dish or glass bottom dish and cultured overnight. The cells were exposed to 10 Gy *γ*‐rays using a Gammacell 40 exactor (Nordion International, Ottawa, Canada). After incubation for 24, 72, and 120 h, the cells were collected, and several cellular senescence markers were evaluated by immunoblotting (details are described below). Characteristic morphological changes of senescent cells were detected by IX63 microscope (Olympus, Tokyo, Japan). The presence of senescent cells was also determined using SPiDER‐βGal (Dojindo; Kumamoto, Japan) to detect β‐galactosidase by microscopy. The up‐regulation of β‐galactosidase in senescent cells was detected by using a senescence‐associated β‐galactosidase staining kit (#9860, Cell Signaling Technology; Danvers, MA, USA) according to the manufacturer's instructions.

### 2.5 Immunoblotting

Cells were collected and lysed with RIPA buffer (Cell Signaling Technology). After centrifugation, the supernatants were collected, four‐fold concentrated NuPAGE™ LDS Sample Buffer was added and samples were boiled for 5 min. The proteins in cell samples were then separated by SDS‐PAGE and transferred onto a PVDF membrane. The membrane was blocked with Bullet Blocking One (Nacalai Tesque; Kyoto, Japan) and probed with the relevant primary antibody. After subsequent incubation with HRP‐conjugated secondary antibodies, protein signals were detected with Western Lightning ONE Femto (PerkinElmer; Yokohama, Japan). Image acquisition was performed with a FUSION SOLO S device western blot and chemiluminescence imaging system (Vilber; Collégien, France), and image analysis was conducted using ImageJ software.

### 2.6 In vitro NIR‐PIT under fluorescence microscopy

Cells were seeded onto a glass bottom dish and cultured overnight. After irradiation with *γ*‐rays and incubation for 5 days, the cells were incubated with phenol‐red free media containing Pan‐IR700 or Tra‐IR700 (10 μg/mL) for 1 h at 37°C. To detect the dead cells induced by NIR‐PIT, propidium iodide (PI) was added to the medium immediately before NIR light exposure. NIR irradiation and microscopic analysis were performed using an IX63 microscope with reflected light fluorescence and a filter set consisting of an excitation filter (670.5–745.5 nm). To detect the fluorescence of PI, a filter set consisting of an excitation filter (530–550 nm) and an emission filter (575–625 nm) was used.

### 2.7 Cell viability assay in NIR‐PIT and paclitaxel treatment

Cells were seeded into a 24‐well plate and cultured overnight. The cells were irradiated with 10 Gy *γ*‐rays and incubated for 5 days. Before in vitro NIR‐PIT, cell viability (Pre) was assessed by using a Cell Counting Kit‐8 (Dojindo) according to the manufacturer's instructions. Then, the cells were incubated with phenol‐red free media containing Pan‐IR700 or Tra‐IR700 (10 μg/mL) for 1 h at 37°C. Cells were illuminated with a laser‐emitting light at a 690 nm continuous wave laser (MLL‐III‐690, CNI Technology, Changchun, China) with output timing controllable by a TTL control unit (Shimadzu Corporation, Kyoto, Japan) at a power density of 50 mW/cm^2^ as measured with an optical power meter (PM 100, Thorlabs, Newton, NJ, USA). After incubation for 3 h, cell viability (Post) was monitored using a Cell Counting Kit‐8. The absorbance of each sample was measured at 450 nm with a microplate reader (GloMax Discover; Promega, Madison, WI, USA). For paclitaxel treatment, non‐senescent and senescent A549 cells were treated with paclitaxel (Fujifilm Wako Pure Chemical Industries) for 48 h. Cell viability was assessed by Cell Counting Kit‐8.

### 2.8 Statistical analysis

All results are expressed as mean ± standard deviation (SD) of the mean. Statistical analysis was carried out using GraphPad Prism 8.0 (GraphPad Software, Inc, San Diego, CA, USA). Comparison of two groups were performed using Student's *t*‐tests. For multiple comparisons, a Tukey–Kramer test was used. The minimum level of significance was set at *p* < 0.05.

## 3 RESULTS

### 3.1 Induction of cellular senescence in cancer cell lines

**FIGURE 1:**
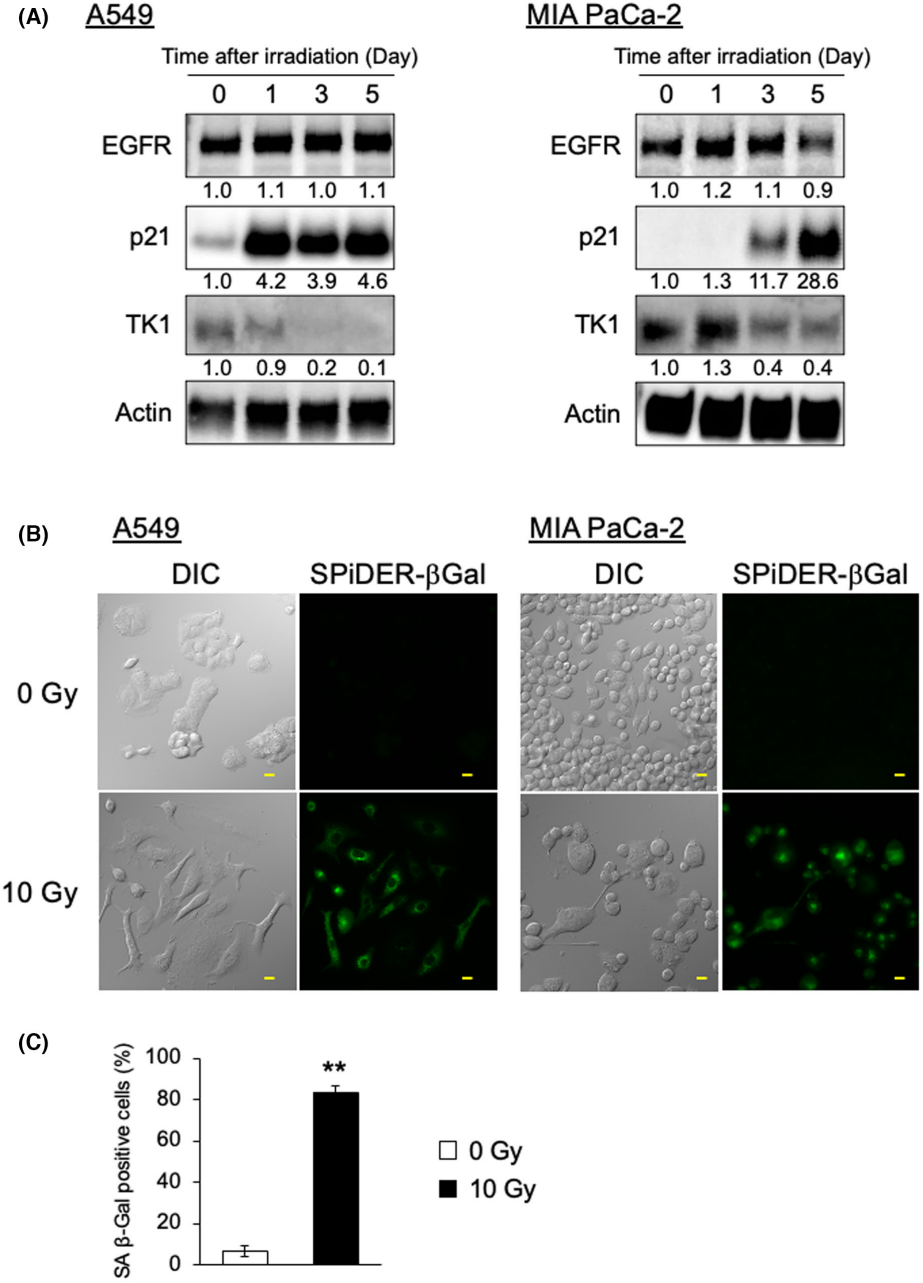
Cellular senescence induced by *γ*‐ray irradiation of A549 and MIA PaCa‐2 cells. (A) The expression profiles of cellular senescence‐related proteins and EGFR were analyzed by immunoblotting. Actin was used as a loading control. Numbers beneath each lane represent densitometric ratios of each protein normalized to that of Day 0. (B) Representative differential interference contrast (DIC) and fluorescence images of A549 and MIA PaCa‐2 cells after irradiation at 10 Gy. SPiDER‐βGal was used for the detection of senescent cells. Scale bar, 20 μm. (C) Senescence associated β‐galactosidase (SA β‐gal) assay of A549 cells after *γ*‐ray irradiation. At least 200 cells were analyzed, and the percentage of SA β‐Gal positive cells was determined. **p < 0.01 versus 0 Gy (Student's *t*‐test). TK1, thymidine kinase 1.

Previous reports suggested that the expression level of EGFR is altered in senescent cells^26^; however, we found it unchanged in A549 and MIA PaCa‐2 cells (Figure 1A). The expression level of p21, a senescence‐related protein, was sufficiently up‐regulated at 5 days after the irradiation of A549 and MIA PaCa‐2 cells (Figure 1A). Moreover, TK1, one of the proliferation markers, was down‐regulated by irradiation in both cell lines. These results indicated that the irradiation with *γ*‐rays induced the long‐term arrest of cellular proliferation. In microscopic experiments, cells showed dramatic morphological changes, including enlargement, flattening, and multinucleation (Figure 1B). In addition, an increased number of A549 and MIA PaCa‐2 cells stained positive for SPiDER‐βGal, the fluorescence probe for senescent cells. A SA‐β‐Gal assay was performed in A549 cells for quantitative evaluation of senescent cells. As shown in Figure 1C, the number of SA‐β‐Gal positive cells were significantly increased by 10 Gy irradiation (Control: 6.8 ± 2.7% vs. 10 Gy: 83.2 ± 3.6%). These results suggested that cellular senescence was definitely induced in tumor cells under our irradiation conditions.

### 3.2 In vitro NIR‐PIT in senescent cancer cells

**FIGURE 2:**
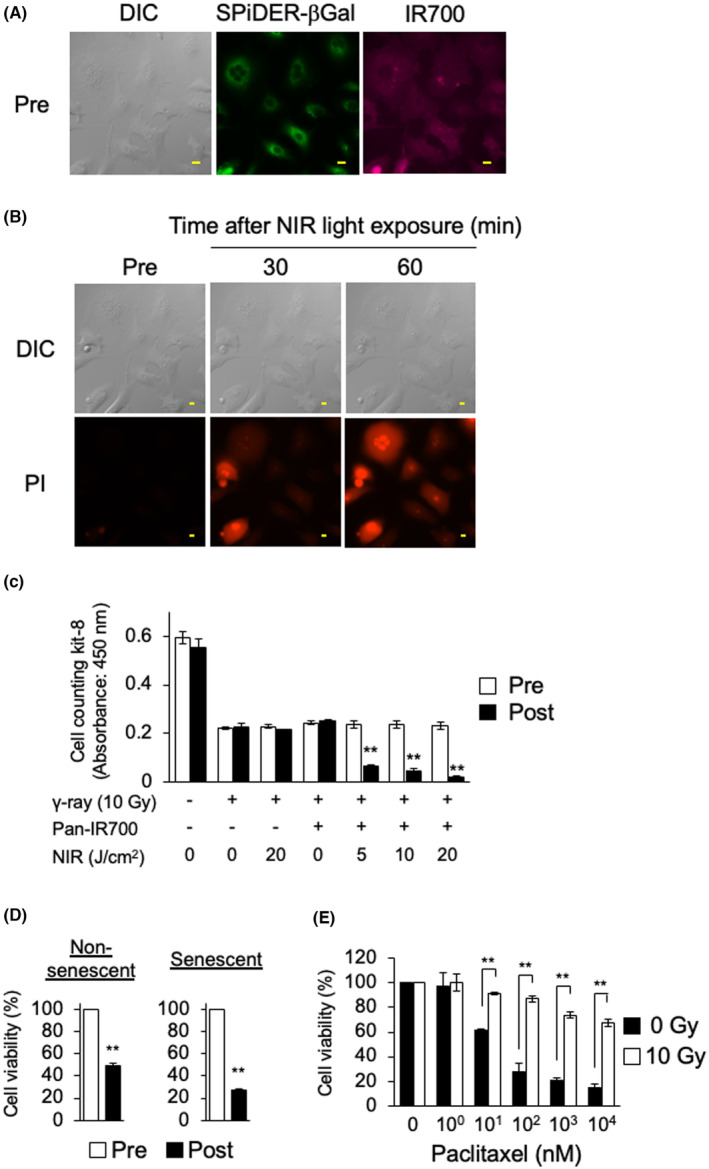
NIR‐PIT induced cell death in senescent A549 cells. (A) Representative DIC (left panel) and fluorescence images. SPiDER‐βGal was used for the detection of senescent cells (middle panel). The binding of Pan‐IR700 in senescent A549 cells was evaluated (right panel). Scale bar, 20 μm. (B) Morphological changes and inflow of propidium iodide (PI) after near‐infrared (NIR) light exposure. Scale bar, 20 μm. (C) Cell viability before (pre) and after (post) treatment. Data are expressed as the mean ± standard deviation of three independent experiments. **p < 0.01 versus Pre for each group (Student's *t*‐test). (D) The cell variability after NIR‐PIT with 5 J/cm^2^ in non‐senescent and senescent A549 cells. **p < 0.01 versus pre for each group (Student's *t*‐test). (E) Cell viability after paclitaxel treatment in non‐senescent and senescent A549 cells. Data are expressed as the mean ± standard deviation of three independent experiments. **p < 0.01 versus 0 Gy Control (Student's *t*‐test).

In vitro NIR‐PIT targeting EGFR was then performed using fluorescence microscopy. As shown in Figure 2A and Figure S1A, Pan‐IR700 bound to the membrane of senescent A549 and MIA PaCa‐2 cells. After NIR light exposure, morphological changes such as bleb formation and swelling were observed. Moreover, the influx of PI into cells was also observed (Figure 2B; Figure S1B) suggesting that cellular membrane integrity was disrupted by NIR‐PIT. To quantitatively assess the therapeutic efficacy of NIR‐PIT, cellular survival before and after NIR‐PIT was compared in irradiated cells (Figure 2C). Cellular survival was not changed by NIR light exposure‐only and Pan‐IR700‐only treatment. In contrast, cellular survival was significantly decreased in a NIR‐light dose‐dependent manner in NIR‐PIT‐treated cells. In addition, the cytotoxic effects of NIR‐PIT in senescent A549 cells tended to be higher than that of non‐senescent cells (Figure 2D). To assess whether senescent cells could be eliminated by conventional chemotherapy, senescent A549 cells were treated with paclitaxel, which induces apoptosis. The sensitivity to paclitaxel was significantly lower in senescent compared to non‐senescent cells (Figure 2E). These results suggested that NIR‐PIT was effective in even radiation‐induced senescent cells with apoptotic stimuli resistance.

### 3.3 In vitro NIR‐PIT in senescent stromal cells

**FIGURE 3:**
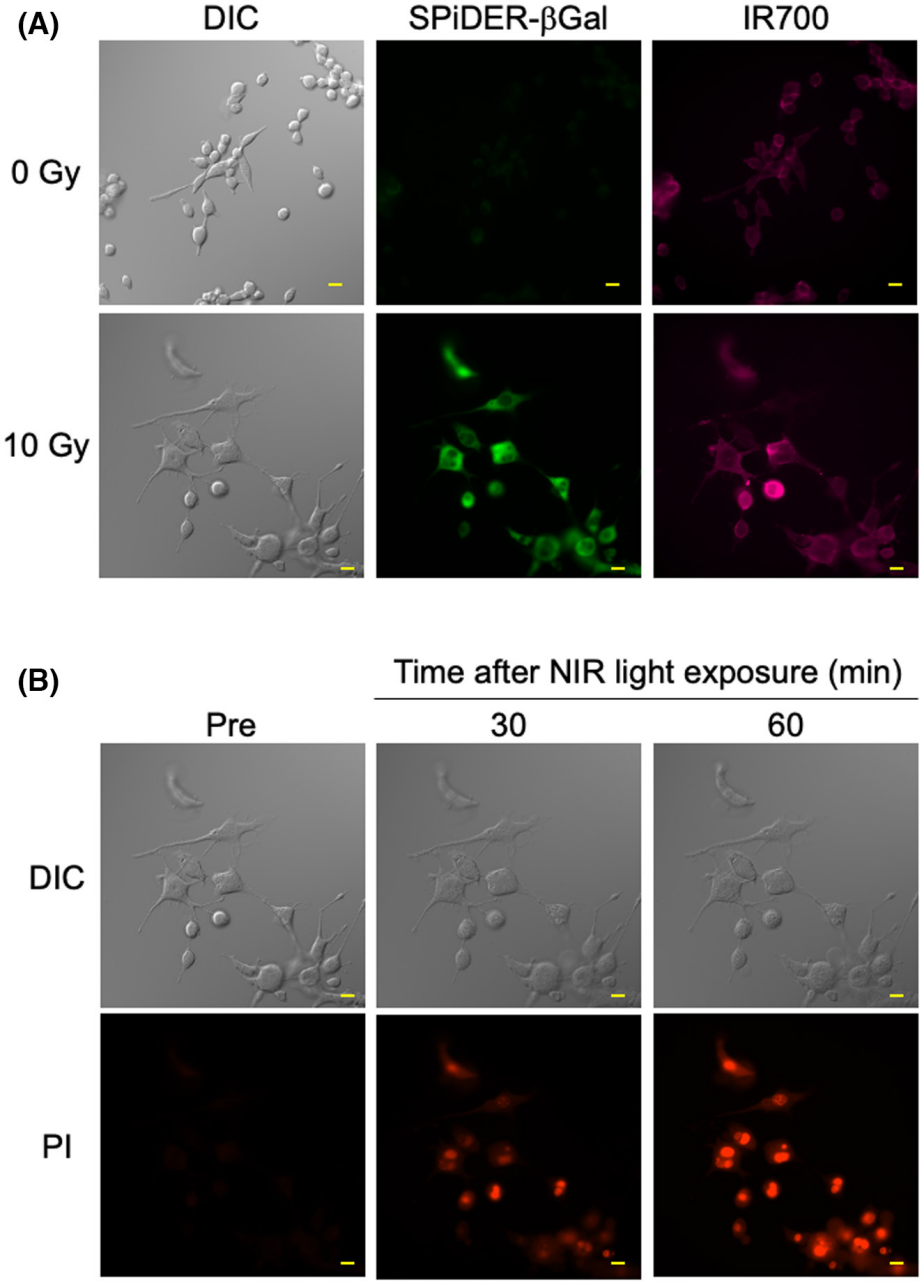
NIR‐PIT induced cell death in senescent 3 T3‐HER2 cells. (A) Representative DIC (left panels) and fluorescence images. SPiDER‐βGal was used for the detection of senescent cells (middle panels). The binding of Tra‐IR700 in senescent 3 T3‐HER2 cells was evaluated (right panels). (B) Morphological changes and inflow of PI after NIR light exposure. Scale bar, 20 μm.

To evaluate the therapeutic efficacy of NIR‐PIT in senescent stromal cells, 3T3‐HER2 cells were irradiated with 10 Gy *γ*‐ray. Five days after irradiation, morphological changes in cells, including enlargement, flattening, and multinucleation, and positive staining with SPiDER‐βGal were observed (Figure 3A). In addition, morphological changes and positive staining with SPiDER‐βGal were also observed in normal human fibroblast WI38 cells after *γ* irradiation (Figure S2A). These results suggested cellular senescence was induced in stromal cells under our irradiation conditions. Morphological changes and PI uptake were observed in both senescent 3T3‐HER2 and WI38 cells after NIR‐PIT (Figure 3B; Figure S2B, respectively), as was also the case for non‐senescent 3T3‐HER2 and WI38 cells (Figure S3). These results suggested that NIR‐PIT was effective in not only tumor cells but also senescent stromal cells.

## 4 DISCUSSION

In NIR‐PIT, severe membrane damage is triggered by photochemical actions. In response, morphological changes, including bleb formation and swelling, and the influx of extracellular fluid are induced, resulting in necrosis.^27^ In the current study, NIR‐PIT targeting EGFR induced morphological changes and PI uptake in senescent cancer cells induced by irradiation with *γ*‐rays (Figure 2; Figure S1). Moreover, cellular viability was decreased in a NIR‐light dose‐dependent manner suggesting that NIR‐PIT may induce cytotoxicity with the same mechanism in senescent cancer cells. Although NIR‐PIT has shown therapeutic effects in a variety of cancer and non‐cancer cells,^25^ no reports exist of studies on senescent cells. One of the characteristics of senescent cells is the upregulation of anti‐apoptotic family proteins, leading to the acquisition of resistance to apoptosis.^28^ This anti‐apoptotic capacity makes it difficult to eliminate senescent cells with conventional therapies. Indeed, in the current study, the sensitivity to paclitaxel was significantly lower in the senescent compared to non‐senescent cells, suggesting that additional chemotherapy for senescent cells would be of little benefit (Figure 2E). However, the primary cytotoxic mechanism in NIR‐PIT is necrosis triggered by plasma membrane damage; thus, cellular intrinsic factors hardly contribute to therapeutic efficacy. Accordingly, NIR‐PIT will be an ideal treatment for senescent tumor cells, which considered to greatly expanding the range of therapeutic application of NIR‐PIT.

NIR‐PIT induced morphological changes and membrane damage in non‐senescent fibroblasts similar to that previously reported for cancer cells (Figure S3).^21^ Importantly, NIR‐PIT induced the same phenomenon in senescent fibroblasts (Figure 3; Figure S3), suggesting it is able to eliminate even senescent stroma cells with the same cytotoxic mechanism. In the current study, NIR‐PIT targeting EGFR was employed for treating senescent cells. CAFs derived from patients with colorectal cancer express EGFR,^29^ suggesting that NIR‐PIT using cetuximab‐IR700 conjugates currently used in clinical practice may eliminate cancer and stroma cells simultaneously regardless of whether they are senescent or not. CAFs are potentially radioresistant; senescent cells remain in tumor tissue after treatment and trigger chronic inflammation that encourages tumor progression.^11^, ^12^, ^13^ Moreover, SASP factors from senescent stroma induces PD‐L1 upregulation in the tumor, T‐cell suppression, and an increase in T‐cell‐suppressive myeloid cells, leading to an immunosuppressive microenvironment.^30^ Notably, NIR‐PIT induces immunogenic cell death that initiates the activation of the adaptive immune response in addition to a direct cytotoxic effect.^31^, ^32^ It is possible that these effects are supported by the unintentional removal of senescent cells. Further verification may lead to a more efficient anti‐cancer strategy.

NIR‐PIT targeting EGFR has been approved in Japan for patients with advanced head and neck cancer, who have previously received radiotherapy, and has shown good therapeutic effects in clinical practice. Previous reports suggested that radiation‐induced senescence positively correlates with radioresistance in head‐and‐neck squamous cell carcinoma cells.^33^ Moreover, the expression of the cellular senescence markers, p16INK4a and ARF, was increased immediately after chemotherapy and remained elevated 12 months after treatment.^34^ Considering these facts, the therapeutic effect of NIR‐PIT in clinical practice may already include the effect of eliminating senescent tumor and stromal cells. However, further investigations are needed to clarify whether therapeutic efficacy is involved in the elimination of senescent cells.

The cytotoxic effects are induced only in conjugates‐bound and NIR‐light‐exposed cells regardless of cellular phenotype. Therefore, NIR‐PIT induced necrosis in both non‐senescent and senescent fibroblasts with expressing target antigen (Figure 3; Figures S2 and S3), which may raise concerns about side effects on normal cells. Although epithelial cells originally express of EGFR, the expression level are much lower than tumor tissue. Thus, antibody‐IR700 mainly accumulates in tumor with high expression of EGFR. In addition, the therapeutic effect depends on the amount of antibody‐IR700 binding to the cell.^35^ Furthermore, the cytotoxic effects of NIR‐PIT are induced only where NIR light is irradiated. Therefore, adverse effects on normal cells are unlikely to occur and no serious side effects have occurred in actual clinical practice.^23^ However, it is vital to identify the membrane protein specifically expressed in senescent cells for less side effect senolysis. In the current study, to obtain the evidence that NIR‐PIT could induce cytotoxic effects even in the senescent cells, EGFR and HER2 were employed as a target for NIR‐PIT since their expression was not changed by cellular senescence. The finding that NIR‐PIT can eliminate senescent cells is implying that various membrane proteins could be targets for treating senescent cells. Recently, several senescent cell surface markers such as beta‐2‐microglobulin and dipeptidyl peptidase 4, were identified and antibody‐dependent cell‐mediated cytotoxicity and antibody‐drug conjugates targeting these proteins have been developed.^36^, ^37^ Thus, developing of NIR‐PIT that targets such proteins can enable the more selective elimination of senescent cells, leading to senolysis by NIR‐PIT in the future.

Several limitations exist in this study. Although various types of senescence inducers have been reported, only *γ*‐rays were employed. Most therapy‐induced cellular senescence is associated with unrepaired DNA damage similar to that of *γ*‐rays; thus, the selection of a senescence inducer may hardly affect the senescent phenotype.^38^ Moreover, the cytotoxic mechanism of NIR‐PIT involves membrane damage and subsequent necrosis; differences in endogenous factors of senescent cells may not affect the therapeutic effects of NIR‐PIT. Second, the elimination of senescent cells by NIR‐PIT was demonstrated only in vitro; it is essential to eventually evaluate this in tumor‐bearing mice. However, since biomarkers for senescent cells have not yet been fully identified, and the detection of senescent cells in vivo is methodologically difficult, it would be difficult to demonstrate an in vivo therapeutic effect on senescent cancer cells. Recently, various imaging probes have been developed and used to detect β‐galactosidase‐expressing senescent cells induced by chemotherapy.^39^, ^40^ Such imaging techniques may provide a solution to this challenge.

In conclusion, NIR‐PIT is effective in eliminating senescent cancer and stroma cells induced by irradiation with *γ*‐rays in vitro. Such results are relevant to the development of novel future anti‐cancer therapy.

## AUTHOR CONTRIBUTIONS

**Daiki Hara:** Methodology (equal); writing – review and editing (equal). **Hirofumi Hanaoka:** Project administration (equal); resources (equal); supervision (equal); writing – review and editing (equal). **Motofumi Suzuki:** Conceptualization (equal); data curation (equal); funding acquisition (equal); investigation (equal); methodology (equal); validation (equal); visualization (equal); writing – original draft (equal). **Hisataka Kobayashi:** Supervision (equal); writing – review and editing (equal).

## FUNDING INFORMATION

This work was supported, in part, by the Japanese Society for the Promotion of Science KAKENHI (Grant number 20 K16813 [MS]).

## CONFLICT OF INTEREST STATEMENT

The authors have no conflict of interest.

## ETHICS STATEMENT

Approval of the research protocol by an Institutional Reviewer Board: Not applicable. Informed Consent Statement: Not applicable. Registry and the Registration No. of the study/trial: Not applicable. Animal Studies: Not applicable.

## Supporting information


Figure S1.

## Data Availability

The data presented in this study is available on request from the corresponding author.
