# Supplementary material for: Elimination of radiation‐induced senescent cancer cells and stromal cells in vitro by near‐infrared photoimmunotherapy
Source: Cancer Med. 2024 Jun 18;13(12):e7381. doi: 10.1002/cam4.7381 (PMC11184651; doi:10.1002/cam4.7381)
Supplement: Supplementary file 1 — Figure S1. [file CAM4-13-e7381-s001.pdf]

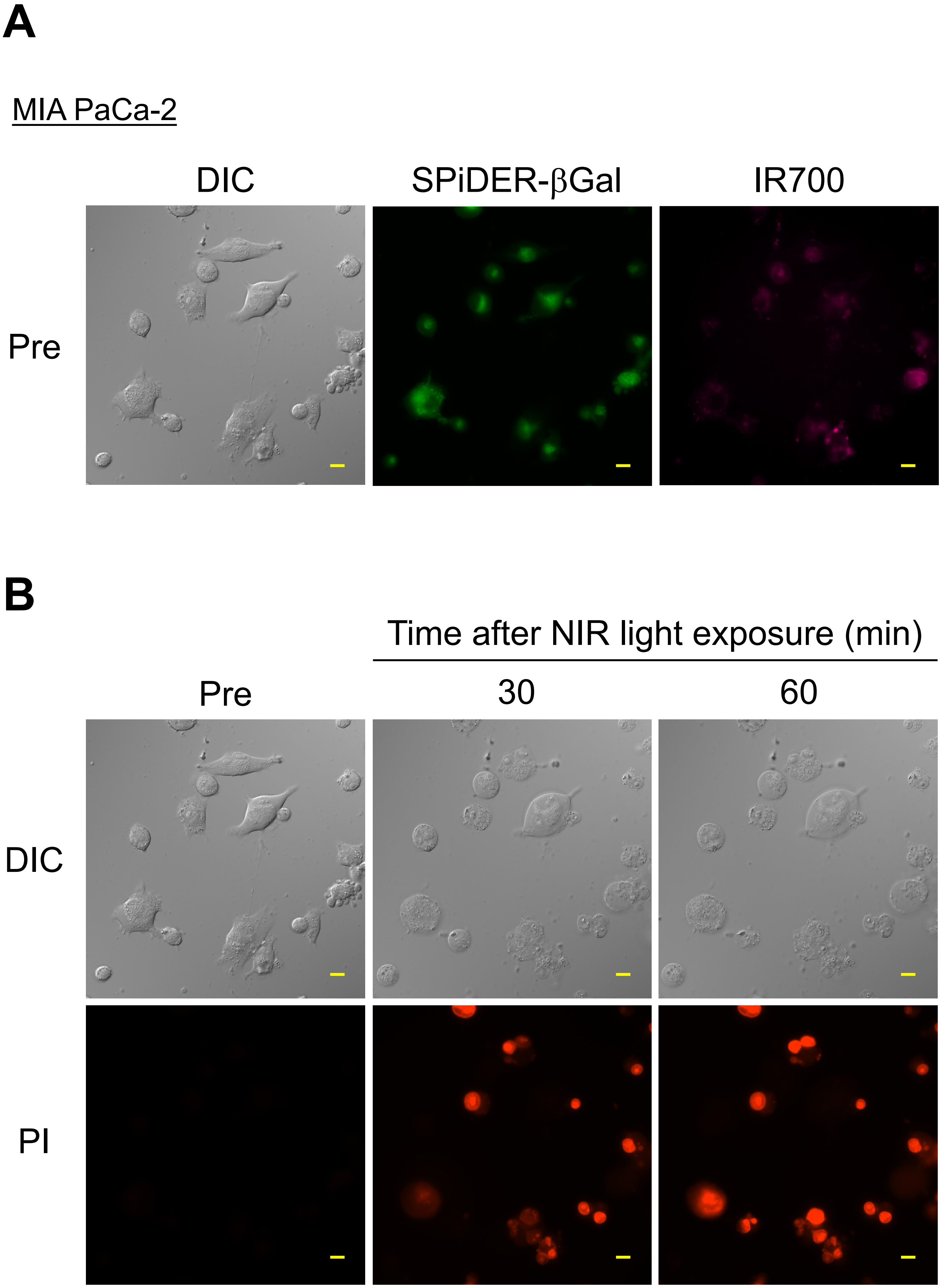

Supplemental figure 1. Suzuki et al.

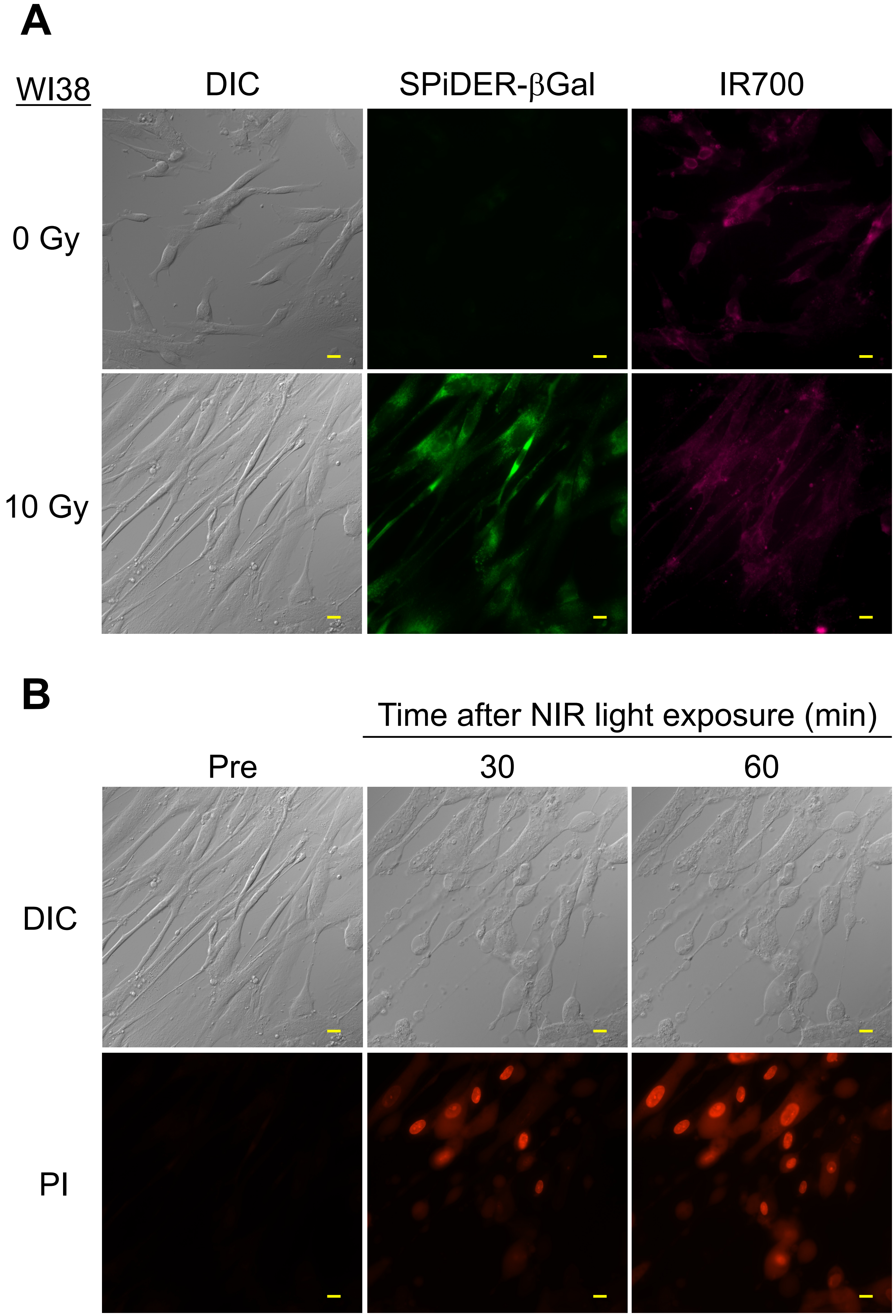

Supplemental figure 2. Suzuki et al.

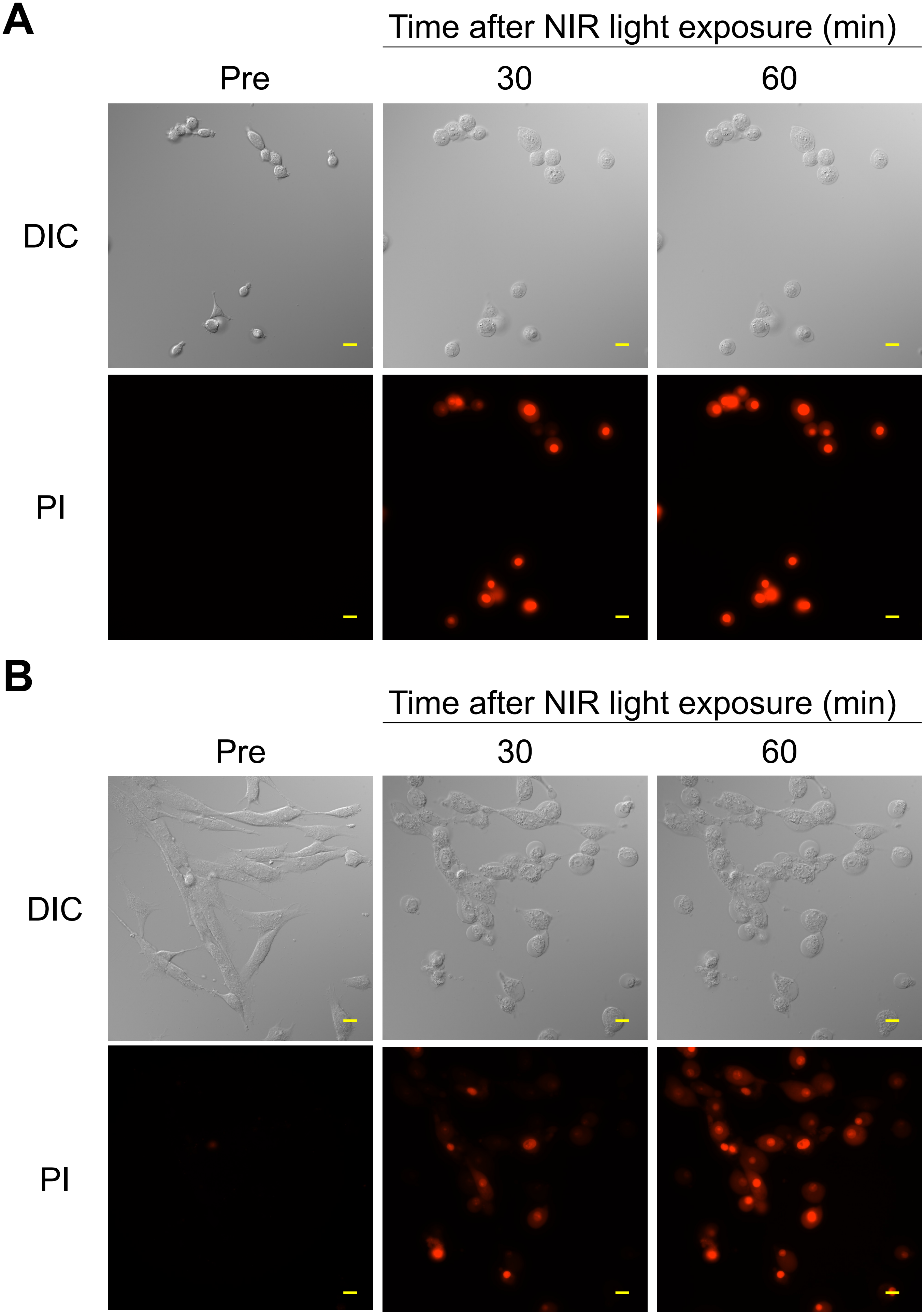

Supplemental figure 3. Suzuki et al.
